# Supplementary material for: Effect of Prior ChAdOx1 COVID-19 Immunisation on T-Cell Responses to ChAdOx1-HBV
Source: Vaccines (Basel). 2024 Jun 9;12(6):644. doi: 10.3390/vaccines12060644 (PMC11209196; doi:10.3390/vaccines12060644)
Supplement: Supplementary file 1 [file vaccines-12-00644-s001.zip › vaccines-2914556-supplementary.pdf]

Supplementary Table S1: Intracellular cytokine staining assay reagents.

Live dead/ Dextramer/Antibody	Dye/Fluorochrome	Clone	Function	Vendor
Zombie Viability dye	NIR	n/a	Dead cells exclusion	Biolegend
**Surface Antibodies**				
CD3	AF700	SK7	Define lineage	Biolegend
CD4	cFluor B548	SK3		Cytek Biosciences
CD8	cFluor V547	SK1		Cytek Biosciences
CCR7	APC/Fire 810	G043H7	CD8/CD4 naïve and memory subsets	Biolegend
CD45RA	BV570	H100		
CD14	Pacific Blue	HCD14	Define lineage (monocytes exclusion)	
CD19	Pacific Blue	HIB19	Define lineage (B cells exclusion)	
**Intracellular Antibodies**				
IFNg	Pe-Cy7	B27	Cytokine response	BD Biosciences
TNFa	BV750	Mab11		BD Biosciences
IL-2	PE	MQ1-17H12		Biolegend
CD107a	BV711	H4A3	CD8 T cell activation	Biolegend
CD154	BV480	TRAP1	CD4 T cell activation	BD Biosciences

Supplementary Table S2: Local and systemic reactogenicity.

		Healthy Participants		CHB Participants		Healthy Participants Covid-19 Vaccinated		Total
		Low Dose (N=5) n (%)	High Dose (N=5) n (%)	Low Dose (N=6) n (%)	High Dose (N=5) n (%)	ChAdOx1 (N=15) n (%)	mRNA (N=11) n (%)	(N=47) n (%)
Any Reaction	n (%)	2 (40.0)	4 (80.0)	3 (50.0)	3 (60.0)	9 (60.0)	9 (81.8)	30 (63.8)
Any Local Reaction	n (%)	1 (20.0)	4 (80.0)	3 (50.0)	3 (60.0)	8 (53.3)	9 (81.8)	28 (59.6)
Swelling	n (%)	0	0	0	0	1 (6.7)	2 (18.2)	3 (6.4)
Redness	n (%)	0	0	0	0	3 (20.0)	1 (9.1)	4 (8.5)
Pain	n (%)	1 (20.0)	4 (80.0)	3 (50.0)	3 (60.0)	5 (33.3)	9 (81.8)	25 (53.2)
Warmth	n (%)	0	2 (40.0)	2 (33.3)	1 (20.0)	0	4 (36.4)	9 (19.1)
Any Systemic Reaction	n (%)	2 (40.0)	4 (80.0)	3 (50.0)	3 (60.0)	6 (40.0)	9 (81.8)	27 (57.4)
Temperature	n (%)	0	1 (20.0)	0	0	0	0	1 (2.1)
Muscle Ache	n (%)	1 (20.0)	4 (80.0)	3 (50.0)	2 (40.0)	3 (20.0)	8 (72.7)	21 (44.7)
Fatigue	n (%)	1 (20.0)	1 (20.0)	3 (50.0)	2 (40.0)	1 (6.7)	5 (45.5)	13 (27.7)
Headache	n (%)	0	1 (20.0)	1 (16.7)	1 (20.0)	0	3 (27.3)	6 (12.8)
Nausea	n (%)	0	0	1 (16.7)	1 (20.0)	0	0	2 (4.3)
Feverishness	n (%)	0	1 (20.0)	2 (33.3)	0	0	0	3 (6.4)
Chills	n (%)	0	1 (20.0)	0	1 (20.0)	1 (6.7)	1 (9.1)	4 (8.5)
Joint Ache	n (%)	0	1 (20.0)	1 (16.7)	1 (20.0)	1 (6.7)	1 (9.1)	5 (10.6)
Malaise	n (%)	0	1 (20.0)	1 (16.7)	1 (20.0)	0	2 (18.2)	5 (10.6)

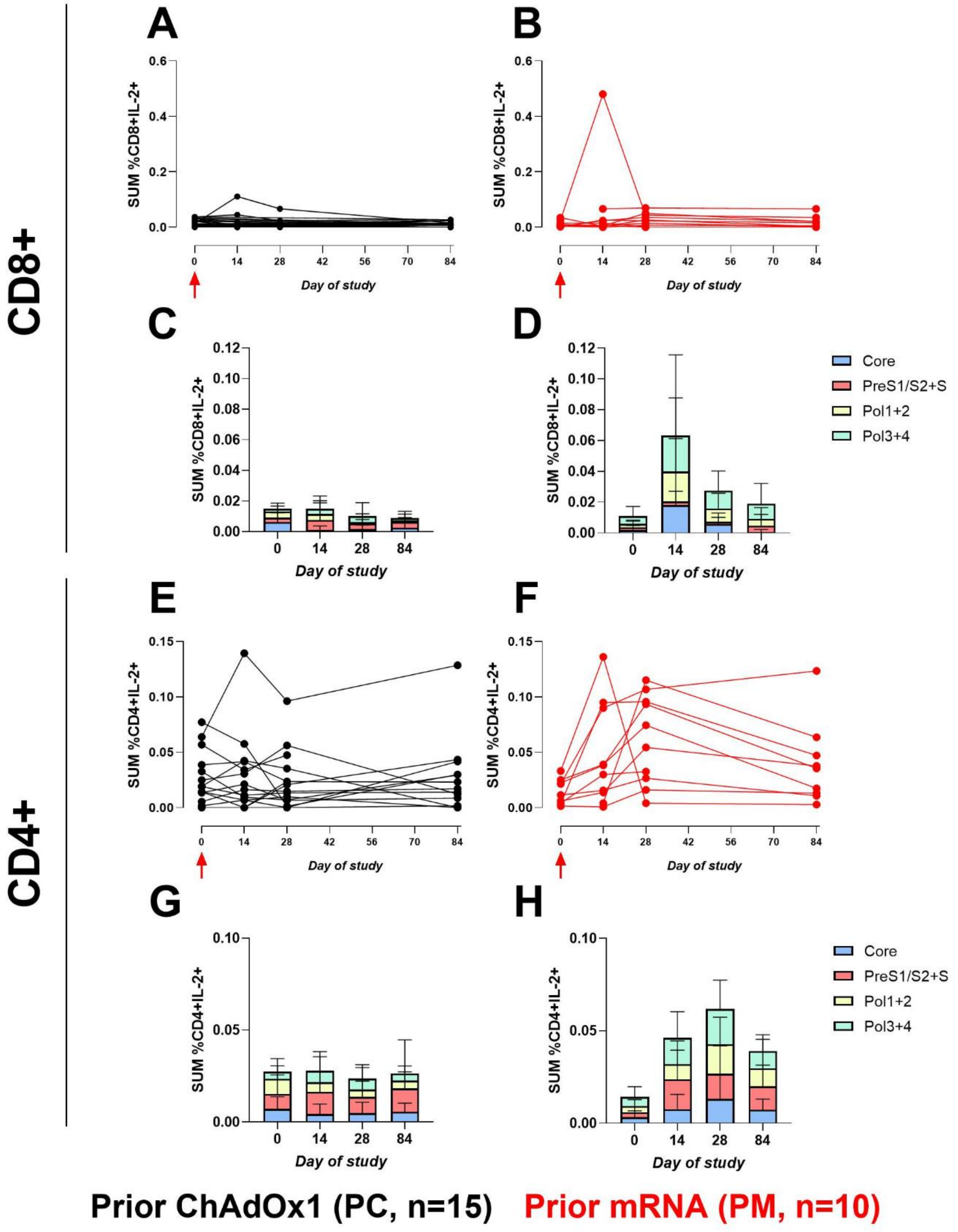

Supplemental Figure S1: Sum of HBV-specific CD8+ or CD4+ IL-2+ ICS responses.

Line graphs represent the sum of CD8+ or CD4+ IL-2+ HBV-specific peptide responses (Core, Pol1+2, Pol3+4, PreS1/S2+S, % of total population) for each participant across the study for PC and PM respectively. Red arrow indicates ChAdOx1-HBV administration. **A**, CD8+, PC and **B**, CD8+, PM, **E**, CD4+, PC and **F**, CD4+, PM. Stacked bar graphs represent the sum of mean responses to HBV-specific peptide pools. **C**, CD8+, PC and **D**, CD8+, PM, **G**, CD4+, PC and **H**, CD4+, PM. Note: difference in scale of the Y-axes between CD8+ and CD4+ plots.

Supplementary Table S3: Median, interquartile range (SFU/10^6 PBMC) and P value (Mann-Whitney) for ELISpot readouts organised by HBV-specific and Hexon response

ELISpot								
	D0		D14		D28		D84	
Cohort	PC	PM	PC	PM	PC	PM	PC	PM
HBV-specific responses								
25th percentile	0	63	60	160	0	140	0	80
Median	32.5	100	193	313	93	317	132	163
75th percentile	120	237	287	783	220	600	271	403
P value (Mann-Whitney)	0.102		0.07		0.0130		0.441	
Hexon responses								
25th percentile	0	0	163	117	0	107	Unknown	Unknown
Median	87.5	0	207	237	233	143	Unknown	Unknown
75th percentile	250	80	296	423	291	300	Unknown	Unknown
P value (Mann-Whitney)	0.10		0.93		0.83		Unknown	

Supplementary Table S4 – S8: Median, interquartile range (%) and P value (Mann-Whitney) for each ICS readout organised by T cell subset (CD8+ or CD4+)

Supplementary Table S4:

IFNγ								
	D0		D14		D28		D84	
Cohort	PC	PM	PC	PM	PC	PM	PC	PM
CD8+								
25th percentile	0.024	0.016	0.007	0.059	0.008	0.083	0.003	0.087
Median	0.034	0.027	0.025	0.143	0.024	0.150	0.040	0.138
75th percentile	0.043	0.068	0.072	0.219	0.087	0.279	0.081	0.225
P value (Mann-Whitney)	0.98		0.013		0.0078		0.010	
CD4+								
25th percentile	0.006	0.005	0.002	0.024	0.006	0.016	0.008	0.013
Median	0.010	0.011	0.012	0.032	0.015	0.040	0.017	0.034
75th percentile	0.024	0.029	0.020	0.068	0.026	0.088	0.030	0.048
P value (Mann-Whitney)	0.89		0.0029		0.0096		0.21	

Supplemental Table S5:

TNFα								
	D0		D14		D28		D84	
Cohort	PC	PM	PC	PM	PC	PM	PC	PM
CD8+								
25th percentile	0.13	0.18	0.21	0.16	0.16	0.17	0.12	0.26
Median	0.22	0.28	0.25	0.42	0.28	0.36	0.26	0.44
75th percentile	0.33	0.37	0.37	0.66	0.37	0.57	0.31	0.75
P value (Mann-Whitney)	0.34		0.44		0.46		0.030	
CD4+								
25th percentile	0.19	0.16	0.15	0.24	0.17	0.21	0.14	0.21
Median	0.25	0.31	0.20	0.34	0.25	0.32	0.22	0.29
75th percentile	0.33	0.42	0.33	0.41	0.33	0.41	0.35	0.40
P value (Mann-Whitney)	0.64		0.10		0.18		0.22	

Supplemental Table S6:

IL-2								
	D0		D14		D28		D84	
Cohort	PC	PM	PC	PM	PC	PM	PC	PM
CD8+								
25th percentile	0.007	0.004	0.000	0.005	0.001	0.008	0.000	0.003
Median	0.011	0.006	0.005	0.011	0.005	0.025	0.007	0.016
75th percentile	0.025	0.015	0.012	0.026	0.010	0.044	0.013	0.021
P value (Mann-Whitney)	0.38		0.20		0.049		0.13	
CD4+								
25th percentile	0.014	0.004	0.007	0.014	0.007	0.027	0.002	0.013
Median	0.019	0.011	0.016	0.034	0.015	0.064	0.017	0.035
75th percentile	0.039	0.022	0.042	0.090	0.035	0.096	0.030	0.047
P value (Mann-Whitney)	0.31		0.34		0.016		0.21	

Supplemental Table S7:

CD107a								
	D0		D14		D28		D84	
Cohort	PC	PM	PC	PM	PC	PM	PC	PM
CD8+								
25th percentile	0.00	0.05	0.01	0.11	0.01	0.13	0.02	0.13
Median	0.05	0.10	0.04	0.26	0.12	0.22	0.12	0.18
75th percentile	0.09	0.33	0.18	0.74	0.24	0.40	0.30	0.38
P value (Mann-Whitney)	0.21		0.0160		0.080		0.32	

Supplemental Table S8:

CD154								
	D0		D14		D28		D84	
Cohort	PC	PM	PC	PM	PC	PM	PC	PM
CD4+								
25th percentile	0.005	0.014	0.008	0.024	0.006	0.041	0.003	0.013
Median	0.027	0.028	0.018	0.049	0.027	0.060	0.022	0.025
75th percentile	0.033	0.037	0.045	0.093	0.059	0.144	0.036	0.058
P value (Mann-Whitney)	0.60		0.062		0.014		0.26	
